# Using nuclear envelope mutations to explore age-related skeletal muscle weakness

**DOI:** 10.1042/CS20190066

**Published:** 2020-08-26

**Authors:** Edmund Battey, Matthew J. Stroud, Julien Ochala

**Affiliations:** 1 Centre of Human and Applied Physiological Sciences, School of Basic and Medical Biosciences, Faculty of Life Sciences and Medicine, King’s College London, London, SE1 1UL, U.K.; 2 British Heart Foundation Centre of Excellence, School of Cardiovascular Medicine and Sciences, King’s College London, London, U.K.; 3 Randall Centre for Cell and Molecular Biophysics, School of Basic and Medical Biosciences, Faculty of Life Sciences and Medicine, Guy's Campus, King's College London, SE1 1UL, U.K.; 4 Department of Biomedical Sciences, University of Copenhagen, Blegdamsvej 3, 2200 Copenhagen N, Denmark

**Keywords:** aging, nuclear envelopes, skeletal muscle

## Abstract

Skeletal muscle weakness is an important determinant of age-related declines in independence and quality of life but its causes remain unclear. Accelerated ageing syndromes such as Hutchinson–Gilford Progerin Syndrome, caused by mutations in genes encoding nuclear envelope proteins, have been extensively studied to aid our understanding of the normal biological ageing process. Like several other pathologies associated with genetic defects to nuclear envelope proteins including Emery–Dreifuss muscular dystrophy, Limb–Girdle muscular dystrophy and congenital muscular dystrophy, these disorders can lead to severe muscle dysfunction. Here, we first describe the structure and function of nuclear envelope proteins, and then review the mechanisms by which mutations in genes encoding nuclear envelope proteins induce premature ageing diseases and muscle pathologies. In doing so, we highlight the potential importance of such genes in processes leading to skeletal muscle weakness in old age.

## Introduction

The human lifespan has increased substantially over the past half-century and this trend is projected to continue well into the 21st century [1]. This extension of the lifespan, however, has not been accompanied by an equivalent extension of the healthspan in old age; instead, morbidity has been extended, and independence and quality of life attenuated [2]. This increasing dependence on healthcare services has associated economic costs. Thus, a ‘managed compression of morbidity’ is necessary to address social and economic issues associated with an extending lifespan [3].

One important aspect of deteriorated healthspan and morbidity is skeletal muscle weakness, which is detrimental for independence and quality of life [4,5]. The causes of such progressive age-related generalised muscle dysfunction remain poorly understood, limiting development of potential therapeutic interventions to improve healthspan (pharmacologically or physically via personalised exercise regimens).

Interestingly, in recent years, premature ageing syndromes caused by genetic defects to nuclear envelope proteins (estimated prevalence of 1:4,000,000 to 1:10,000,000) have been increasingly studied as models to reveal the causes of the normal biological ageing process [6–8]. Among these is the Hutchinson–Gilford Progerin Syndrome (HGPS), which results in severe pathologies including heart disease, arteriosclerosis and an average life expectancy of ∼13 years [9,10]. HGPS and other related disorders such as Emery–Dreifuss muscular dystrophy, Limb–Girdle muscular dystrophy and congenital muscular dystrophy can also lead to severe muscle pathologies resembling muscle weakness in old age, suggesting how modifications in the nuclear envelope may alter skeletal muscle development and function. Such deterioration in skeletal muscle behaviour is often not discussed in the clinical literature, partly because of the severity of the phenotype and the early age of death of patients, which makes the study of skeletal muscle function challenging; hence, its extent remains unclear.

In this review, we start by briefly summarising what is known about nuclear membrane proteins. We then describe the mechanisms by which mutations in genes encoding these nuclear envelope proteins induce premature ageing diseases and muscle pathologies. Finally, we suggest how this knowledge could be used to unveil the key determinants of skeletal muscle weakness in old age.

## The nuclear envelope and mechanotransduction

**Figure 1:**
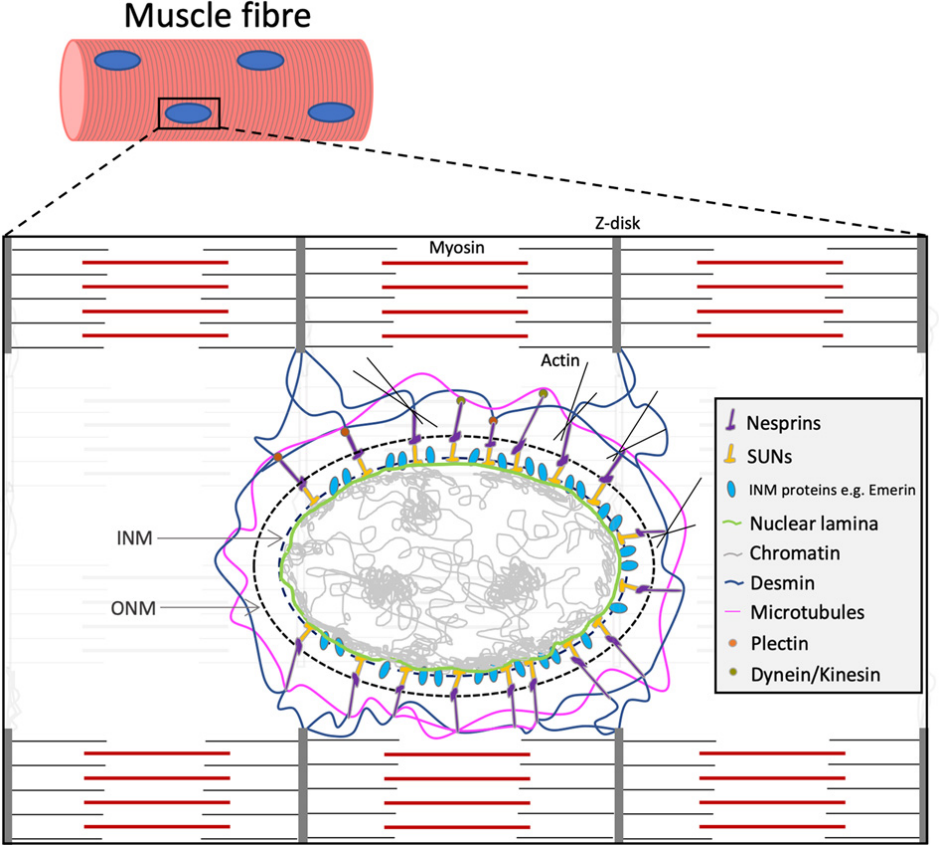
A muscle nucleus connected to sarcomeres through nuclear membrane and cytoskeletal proteins Chromatin (grey) is organised into exposed, transcriptionally active sections (euchromatin) and tightly packed, transcriptionally repressed sections located at nucleoli and the nuclear periphery (heterochromatin). Heterochromatin associates with DNA interaction proteins BAF and HDAC3, which associate with inner nuclear membrane (INM) proteins such as Emerin, and the nuclear lamina, which interacts with SUN 1/2. SUN 1/2 bind to Nesprins to form the LINC complex, which links the nucleus to actin, as well as microtubules and Desmin via Kinesin and Plectin, respectively. Desmin binds to the Z-disk of sarcomeres, completing the connection between nuclei and myofibrils. Through this network of proteins, transcriptional activity is responsive to cytoskeletal changes; INM, inner nuclear membrane, ONM, outer nuclear membrane.

As the site of DNA transcription, the nucleus is responsible for orchestrating cell structure, function and adaptive responses [11]**.** Each nucleus is surrounded by an envelope, termed the nuclear envelope (NE), which sets a barrier between the cytoplasm and nuclear contents and consists of outer and inner nuclear membranes (ONM and INM, respectively) [12]. Some NE and associated proteins physically link the nucleus to the cytoskeleton, providing an interconnected cellular network allowing transduction of physical forces to regulate biochemical signalling and gene expression, a process termed mechanotransduction [13–16]. One group of such proteins is the LInker of Nucleoskeleton and Cytoskeleton (LINC) complex, made up of Nuclear Envelope SPectRIN repeat proteins (Nesprins) and Sad1 and UNC-84 domain containing (SUN) proteins [17]. Nesprins reside in the ONM, extend into the cytoplasm and associate with cytoskeletal proteins such as actin, microtubules and desmin; Nesprins are therefore sensitive to changes in cytoskeletal forces [17–20] (Figure 1). The Nesprin-related link to the nucleus is continued by SUN proteins, in which the SUN domain localises to the perinuclear space between the ONM and INM and binds to the KASH (Klarsicht, ANC-1, Syne Homology) domain of Nesprins; and the nucleoplasmic facing N-terminus binds to the nuclear lamina [19,21–23]. The lamina is a meshwork of intermediate filaments that line the INM and tether chromatin to the nuclear periphery, interact with transcription factors to regulate DNA transcription and regulate several signalling pathways [24–28].

The nuclear lamina also associates with several other INM proteins which interact with proteins that bind to chromatin [29–31]. For example, Emerin, a member of the Lamina-associated polypeptide 2, Emerin, MAN1 (LEM) domain-containing family interacts with DNA through the chromatin-associated Barrier to Autointegration Factor (BAF) [32–35]. Emerin also interacts with Histone Deacetylase 3 (HDAC3), a part of the nuclear co-repressor complex which is responsible for the deacetylation of histones to repress gene expression [36,37]. The interaction between Emerin and HDAC3 may thereby control the expression of muscle differentiation-promoting factors MyoD, Myf5 and Pax7 [36,38] (Figure 1). In addition, LEM domain proteins regulate signalling pathways and transcription factor activity independently of chromatin reorganisation [39–41]. In this way, gene expression is tightly controlled by cytoskeletal forces that are transmitted through the LINC complex, nuclear lamina and associated NE proteins to alter chromatin organisation, transcription factor activity and signalling pathways [42–47].

## Mutations in nuclear envelope proteins and related genetic diseases

Hutchinson–Gilford Progerin Syndrome (HGPS) is the most common premature ageing disorder [9,10]. HGPS is notably associated with mutations in the *LMNA* gene, which encodes the nuclear lamina components Lamin A/C, and the *ZMPSTE24* gene, which encodes the zinc metalloprotease 24 enzyme essential for Lamin A/C maturation [8,48]. These mutations disrupt Lamin A expression and function in nuclei of all cell types and can lead to altered forms known as Progerin or Prelamin A [8,49–52]. Mouse models of premature ageing where Lamin A/C and Prelamin A contents are modulated and are able to recapitulate patient phenotypes (i.e. cardiac and arteriosclerosis problems) as well as causing striking generalised skeletal muscle weakness [6,10,53–55]. Indeed, muscle-specific overexpression of human Progerin in mice significantly decreased muscle mass and myofibre size and halved grip strength [55]. Similarly, absence of the *ZMPSTE24* gene, resulting in the inability to form mature Lamin A from Prelamin A, leads to weaker and atrophic lower limb muscles and a reduction in the intrinsic force-generating capacity of myofibres and myofilaments [6,53].

Mutations in the *LMNA* gene also cause diseases not related to HGPS, collectively referred to as laminopathies, such as Emery–Dreifuss muscular dystrophy (EDMD), Limb–Girdle muscular dystrophy and *LMNA*-congenital muscular dystrophy. These are relatively well characterised clinically by skeletal muscular dystrophies and cardiomyopathies [56–61]. Other missense and nonsense mutations in genes encoding LINC complex and associated NE proteins have been identified in humans and have similar cardiac and skeletal muscle phenotypes. For instance, mutations have been found in *EMD*, encoding Emerin; *TMEM43* encoding Luma; and *SYNE1* and *SYNE2*, encoding Nesprin-1 and Nesprin-2, respectively [62–71].

## Mechanisms underlying skeletal muscle weakness associated with mutations in genes encoding LINC complex and associated proteins

### Lamin A-related disruption in nuclear architecture and mechanotransduction

The mechanisms of skeletal muscle dysfunction associated with mutations in genes for NE proteins are complex and in-depth mechanistic studies are currently lacking. The absence of Lamin A/C is usually characterised by aberrant nuclear morphology, nuclear mechanics and mechanotransduction [42,48,61,72]. Lamin A/C-deficient fibroblasts display increased nuclear deformation and altered expression of mechanosensitive genes *egr-1* and *iex-1* in response to mechanical strain and altered NF-κB signalling [42]. When myoblasts from Lamin A-deficient mice are cultured to form *in vitro* muscle fibres, nuclear deformations, elongation, and rupture are evident and accompanied by DNA damage [73]. Additionally, cultured myoblasts from a human patient with a muscular dystrophy-causing mutation in the *LMNA* gene exhibit altered nuclear morphology, gene expression and increased cellular senescence [74].

Recently, it was shown that *LMNA*-related congenital muscular dystrophy patient fibroblasts and mouse myoblasts displayed altered Lamin A/C localisation, associated with altered expression and localisation of nuclear envelope proteins [61]. In contrast with control and EDMD cells, Lamin A/C was predominantly localised in the nucleoplasm, rather than forming the nuclear lamina at the nuclear periphery. Absence of peripheral Lamin A/C and its accumulation in the nucleoplasm was associated with attenuated expression and mislocalisation of several nuclear envelope transmembrane proteins, and mislocalisation of Nesprin-1α. The authors postulated that the Lamin A/C mislocalisation and associated misregulation of nuclear envelope proteins may explain the greater severity of *LMNA*-related congenital muscular dystrophy compared with EDMD [61] (Figure 3).

**Figure 2:**
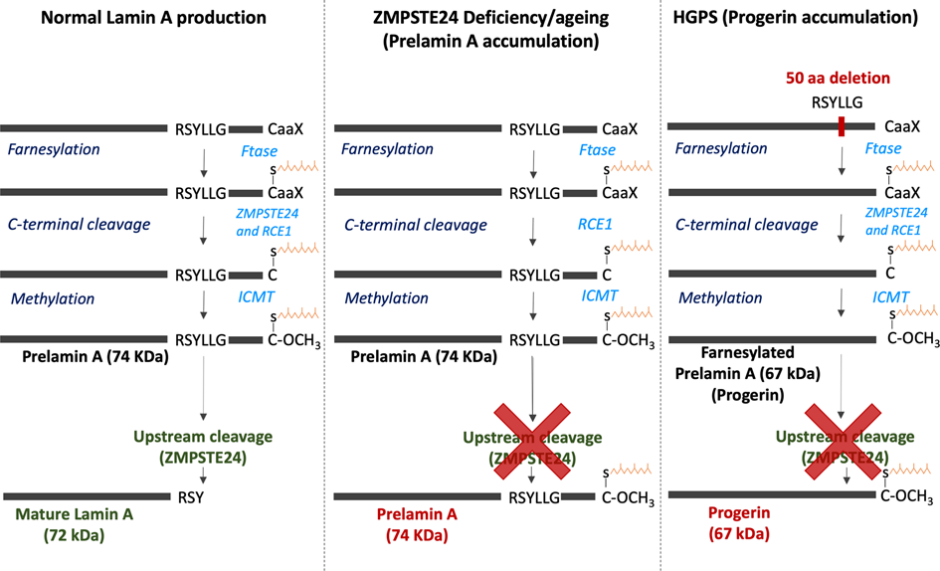
Production of Lamin A, Progerin and Prelamin A from the *LMNA* gene In normal cells, a series of post-translational modifications occurs to form Prelamin A, before cleavage by ZMPSTE24 to produce mature Lamin A. In ZMPSTE24-deficient cells, Prelamin A cannot be cleaved, leading to accumulation of this premature form of Lamin A. In Hutchinson–Gilford progerin syndrome (HGPS) cells, a 50 amino acid deletion removes the site where cleavage by ZMPSTE24 occurs, leading to accumulation of mutant farnesylated Prelamin A, named Progerin. Modified, with permission, from [52]. Enzymes required for the modification steps are in light blue.

Cells expressing Progerin, a truncated, permanently farnesylated form of Lamin A, which lacks the cleavage site for the enzyme *ZMPSTE24* to generate mature Lamin A, show evidence of impaired mechanotransduction, although the specific effects on skeletal muscle cells remain to be determined (Figure 2). Nuclei from Progerin-expressing fibroblasts display aberrant morphology, increased mechanical sensitivity and stiffness leading to senescence and cell death, indicating altered mechanotransduction [48,75]. Interestingly, it has been shown that replacement of mature Lamin A/C by Progerin in fibroblasts and iPSC-derived smooth muscle cells directly triggers premature senescence upon cell differentiation [76,77]. Progerin overexpression has been achieved in human myogenic cells, but whether they displayed a phenotype was not investigated [78].

Cells accumulating Prelamin A, a permanently farnesylated premature form of Lamin A, also exhibit aberrant nuclear morphology in fibroblasts and cardiomyocytes [54,79,80] (Figure 2). It is worth noting altered nuclear morphology has not directly been shown in skeletal muscle and this is an area for further research. However, aberrant nuclear morphology is associated with muscle degeneration, altered transcriptional activity, impaired contractility and muscle weakness, suggesting the accelerated ageing phenotype observed in HGPS fibroblasts could also occur in skeletal muscle cells [6,53,54,81]. A potential mechanism for this muscle weakness is reduced myonuclear number, resulting in a volume of cytoplasm too large for the transcriptional capability of each nucleus (termed the myonuclear domain theory, see [77–79,82–84]). Indeed, reduced myonuclear number was associated with reduced transcriptional activity and myosin content in mice muscle fibres with Prelamin A accumulation [53]. To compound the reduction in myonuclear number, altered nuclear integrity may contribute to defective mechanotransduction, further impairing the transcription of contractile proteins.

### Lamin A-related alteration in Ca^2+^ metabolism

Recently, it was reported that Lamin A and Progerin interact with endoplasmic reticulum based proteins involved in Ca^2+^ transport and alter Ca^2+^ metabolism [55]. Immunoprecipitation in HGPS fibroblasts has shown that both Lamin A and Progerin interact with Sarcolipin, a protein involved in thermogenesis and Ca^2+^ homeostasis; however, Progerin binding is more potent [55,85,86]. Overexpression of Progerin in C2C12 myoblasts resulted in elevated cytosolic Ca^2+^ concentration and altered control of store operated Ca^2+^ entry [55]. Strikingly, mice lacking Lamin A/C upregulate Sarcolipin and skeletal muscle in mice expressing human Progerin have markedly altered Sarcolipin function and Ca^2+^ metabolism, together with ruffled nuclear morphology [55]. Since Ca^2+^ is essential component of muscle contraction, these studies implicate a role of Ca^2+^ metabolism in striated muscle tissue defects caused by mutations in the *LMNA* gene, an area which requires further research [87].

### LINC complex protein-related changes in nuclear behaviour and mechanotransduction

As mentioned earlier, the LINC complex and its associated NE proteins are involved in muscle pathologies and may contribute to skeletal muscle defects with age. For example, fibroblasts expressing muscular dystrophy-associated variants of SUN1 and SUN2 have significant nuclear mispositioning, and this has also been shown in C2C12 myotubes expressing SUN1 variants [88]. In myotubes generated from a human patient with heterozygous SUN1 mutations, myonuclear organisation was similarly defective [88]. In fibroblasts from EDMD patients with Nesprin-1 and -2 mutations, nuclei exhibit aberrant morphologies [66]. Additionally, Nesprin-1 mutant C2C12 cells have defects in myoblast differentiation, specifically reduced fusion index, and down-regulated expression of myogenic transcription factors MyoD and Myogenin, and Myosin Heavy Chain [67]. Ablation of both Nesprin-1 and the muscle-specific isoform, Nesprin-1α2, affects expression of muscle differentiation genes, nuclear number and their positioning, increasing the myonuclear domain and impairing contractile protein expression [53,89–93].

Similarly, Emerin-deficient fibroblasts exhibit abnormal nuclear shape, stability and defective mechanotransduction [72]. In C2C12 cells, Emerin has been shown to interact with A- and B-type lamins, influencing nuclear architecture and stability and ultimately compromising mechanotransduction [94]. Furthermore, loss of Emerin in mice results in nuclear fragility, MAPK signalling activation and delayed induction of MyoD-related genes during muscle regeneration, although overall muscle function and regeneration were minimally affected [27,38,95,96]. The lack of effect on muscle function in Emerin-null mice is unlike the human EDMD phenotype, and may be explained by Lamina-associated polypeptide 1 (LAP1) compensation; LAP1 is highly expressed in mouse compared with human skeletal muscle, and reducing LAP1 expression in Emerin-null mice induced muscle abnormalities [97].

Thus, alterations to Emerin, Nesprin-1/2 and SUN1 in striated muscle tissue can result in nuclear mislocalisation, altered nuclear morphology and mechanotransduction, and impaired development and function. These findings emphasise the role of the LINC complex and associated NE proteins in muscle structure and function, and may therefore have implications for normal physiological ageing, discussed next.

## Implications for normal muscle ageing

**Figure 3:**
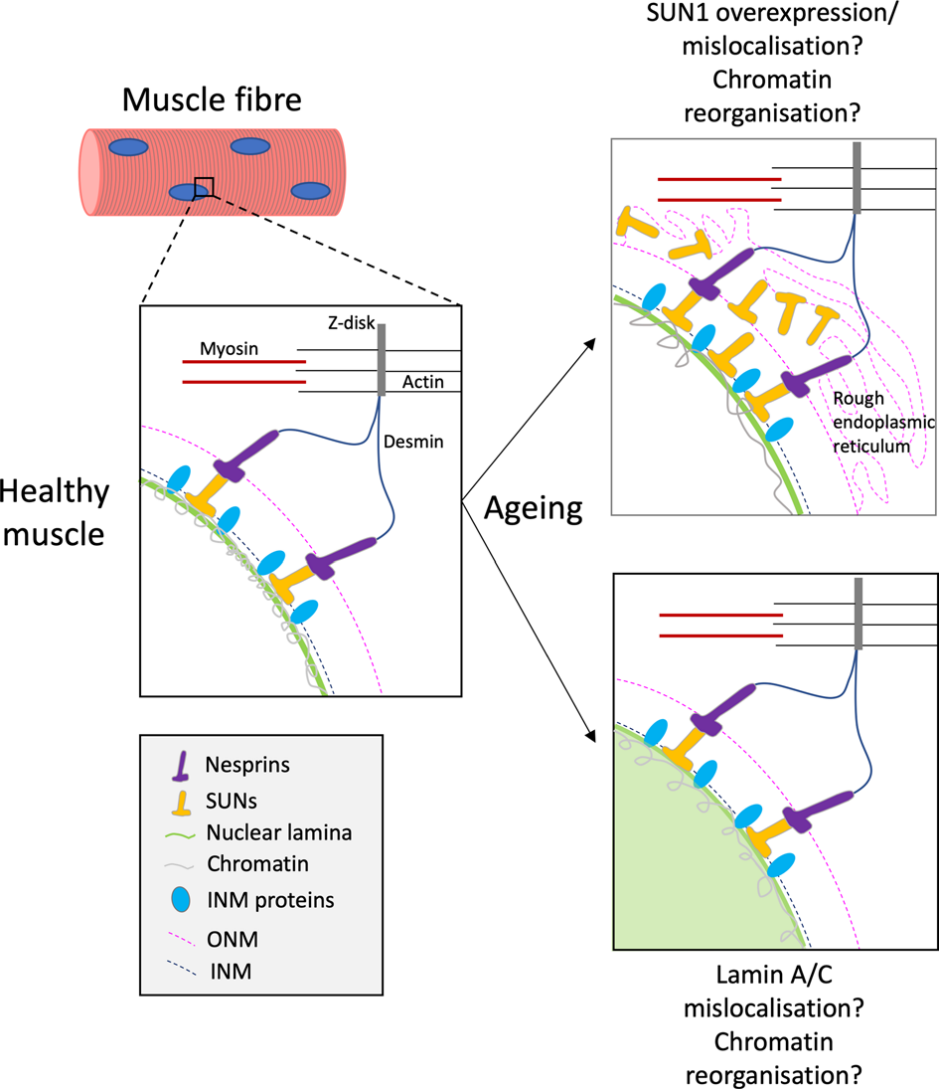
Hypothetical age-related defects in mechanotransduction in skeletal muscle fibres (**Left panel**) In young, healthy skeletal muscle fibres, the LINC complex and its associated nuclear envelope proteins effectively transduce cytoskeletal forces to the nucleus to regulate signalling pathways, normal chromatin organisation and gene expression. (**Right panel**) In aged skeletal muscle, the content or distribution of the LINC complex and its associated proteins may be altered, leading to defective mechanotransduction and a gene transcription that may affect expression of contractile proteins.

The above evidence suggests that alterations in the cytoskeletal-nuclear network could result in structural changes to the nucleus and impact gene expression in skeletal muscle. With age, compromised nuclear integrity, through alterations to LINC complex and associated proteins, may impair mechanotransduction. This may in turn lead to reduced expression of contractile proteins, resulting in a loss of muscle mass and function with age (Figure 3). To date, the only study which has investigated LINC complex proteins in human ageing showed SUN1 levels were increased in fibroblasts from older (84–91 years old) compared with pre-pubescent (3–10 years old) individuals [98]. In line with these data, SUN1 accumulation in *LMNA* mutant mouse fibroblasts and human HGPS patient fibroblasts was associated with nuclear defects and cellular senescence, which were corrected upon reduction of the accumulated SUN1 [99]. Accumulation of SUN1 in human skeletal muscle may strengthen the association between the nucleus and the cytoskeleton, resulting in oversensitivity to cytoskeletal forces thereby causing aberrant mechanotransduction and negatively impacting cell function [98]. Alternatively, SUN1 mislocalisation, as shown in *LMNA^−/-^*fibroblasts, could impair force transmission from the cytoskeleton to the nucleus [99]. Thus, altered SUN1 expression or distribution in skeletal muscle could impair mechanotransduction, elevating atrophic gene expression and suppressing the expression of genes encoding contractile proteins.

## Future research

Current studies mainly focus on alterations to LINC complex components in fibroblasts. Future work using human skeletal muscle biopsies from young and old individuals will be pertinent to investigate mechanisms relating to both skeletal muscle function and dysfunction. Specifically:

1. Establishing whether protein levels and localisation of LINC complex and NE proteins are altered in skeletal muscle from young and old adults, to reveal potential associations between NE function and skeletal muscle ageing.
2. Understanding the effects of exercise on nuclear shape and LINC complex function.
3. Elucidating functional changes to nuclear mechanics in human skeletal muscle to provide insight into the role of NE proteins in aged skeletal muscle.

Furthermore, approaches to model muscular dystrophy in culture using cells from patients to create induced-pluripotent stem cell-derived artificial 3D skeletal muscle may shed new light on pathophysiological mechanisms underlying nuclear envelopathies [100–102].

## Concluding remarks

In this review, we have discussed the mechanisms by which mutations in genes encoding NE proteins induce premature ageing diseases and muscle pathologies, and suggested how this information may be used to unveil the key determinants of muscle weakness in old age. In these diseases, mutations in the LINC complex and its associated proteins cause aberrant nuclear morphology, defective mechanotransduction and muscle weakness. This has been extensively demonstrated in mouse and human fibroblasts, but to a limited degree in muscle cells. Whether changes to NE proteins contribute to defective mechanotransduction and muscle weakness in normal but aged muscle is understudied and is an exciting avenue for future research (Figure 3).

## Open Access

Open access for this article was enabled by the participation of King’s College London in an all-inclusive *Read & Publish* pilot with Portland Press and the Biochemical Society under a transformative agreement with JISC.

## Abbreviations

BAF: barrier to autointegration factor
EDMD: Emery–Dreifuss muscular dystrophy
HDAC3: histone deacetylase 3
HGPS: Hutchinson–Gilford progerin syndrome
INM: inner nuclear membrane
KASH: Klarsicht, ANC-1, Syne Homology
LAP1: lamina-associated polypetide 1
LEM: lamina-associated polypeptide 2, Emerin, MAN1
LINC: linker of nucleoskeleton and cytoskeleton
NE: nuclear envelope
Nesprin: nuclear envelope spectrin repeat proteins
ONM: outer nuclear membrane
SUN: Sad1 and UNC-84 domain containing
